# SWIFT clustering analysis of intracellular cytokine staining flow cytometry data of the HVTN 105 vaccine trial reveals high frequencies of HIV-specific CD4+ T cell responses and associations with humoral responses

**DOI:** 10.3389/fimmu.2024.1347926

**Published:** 2024-06-06

**Authors:** Tim R. Mosmann, Jonathan A. Rebhahn, Stephen C. De Rosa, Michael C. Keefer, M. Juliana McElrath, Nadine G. Rouphael, Giuseppe Pantaleo, Peter B. Gilbert, Lawrence Corey, James J. Kobie, Juilee Thakar

**Affiliations:** ^1^ David H. Smith Center for Vaccine Biology and Immunology, University of Rochester Medical Center, Rochester, NY, United States; ^2^ Vaccine and Infectious Disease Division, Fred Hutchinson Cancer Center, Seattle, WA, United States; ^3^ Department of Medicine, University of Rochester School of Medicine & Dentistry, Rochester, NY, United States; ^4^ Hope Clinic of the Emory Vaccine Center, Division of Infectious Diseases, Emory University, Atlanta, GA, United States; ^5^ Service of Immunology and Allergy, Department of Medicine, Lausanne University Hospital and University of Lausanne, Lausanne, Switzerland; ^6^ Swiss Vaccine Research Institute, Lausanne University Hospital and University of Lausanne, Lausanne, Switzerland; ^7^ Department of Medicine, University of Alabama at Birmingham, Birmingham, AL, United States; ^8^ Department of Microbiology and Immunology, University of Rochester Medical Center, Rochester, NY, United States

**Keywords:** HIV - human immunodeficiency virus, vaccine trial, reanalysis, algorithmic flow cytometry analysis, T cell response, T cell antibody correlation

## Abstract

**Introduction:**

The HVTN 105 vaccine clinical trial tested four combinations of two immunogens - the DNA vaccine DNA-HIV-PT123, and the protein vaccine AIDSVAX B/E. All combinations induced substantial antibody and CD4+ T cell responses in many participants. We have now re-examined the intracellular cytokine staining flow cytometry data using the high-resolution SWIFT clustering algorithm, which is very effective for enumerating rare populations such as antigen-responsive T cells, and also determined correlations between the antibody and T cell responses.

**Methods:**

Flow cytometry samples across all the analysis batches were registered using the swiftReg registration tool, which reduces batch variation without compromising biological variation. Registered data were clustered using the SWIFT algorithm, and cluster template competition was used to identify clusters of antigen-responsive T cells and to separate these from constitutive cytokine producing cell clusters.

**Results:**

Registration strongly reduced batch variation among batches analyzed across several months. This in-depth clustering analysis identified a greater proportion of responders than the original analysis. A subset of antigen-responsive clusters producing IL-21 was identified. The cytokine patterns in each vaccine group were related to the type of vaccine – protein antigens tended to induce more cells producing IL-2 but not IFN-γ, whereas DNA vaccines tended to induce more IL-2+ IFN-γ+ CD4 T cells. Several significant correlations were identified between specific antibody responses and antigen-responsive T cell clusters. The best correlations were not necessarily observed with the strongest antibody or T cell responses.

**Conclusion:**

In the complex HVTN105 dataset, alternative analysis methods increased sensitivity of the detection of antigen-specific T cells; increased the number of identified vaccine responders; identified a small IL-21-producing T cell population; and demonstrated significant correlations between specific T cell populations and serum antibody responses. Multiple analysis strategies may be valuable for extracting the most information from large, complex studies.

## Introduction

The HIV Vaccine Trials Network (HVTN) 105 phase I trial (ClinicalTrials.gov NCT02207920) was designed to build on the encouraging results of the RV144 “Thai Trial” HIV vaccine efficacy trial which demonstrated modest protection from HIV infection (1). RV144 immunization included AIDSVAX B/E consisting of clade B MN gp120 and clade E A244 gp120 proteins in alum given following priming immunizations with a canarypox vector vaccine. HVTN 105 investigated the preventative vaccine strategy of priming with DNA-HIV-PT123 which consisted of 3 plasmids encoding clade C ZM96 gag, clade C ZM96 gp140, and clade C CN54 pol-nef followed by boosting with AIDSVAX B/E in four treatment groups of healthy HIV-1 negative individuals at low risk of HIV acquisition to determine which strategy would best elicit favorable HIV-specific antibody and T cell responses (2). DNA vaccines are thermostable, are relatively straightforward to manufacture, and provide more flexibility for vaccine design through formulation of multiple plasmids containing different HIV components and/or adjuvants in a single injection.

**Figure 1:**
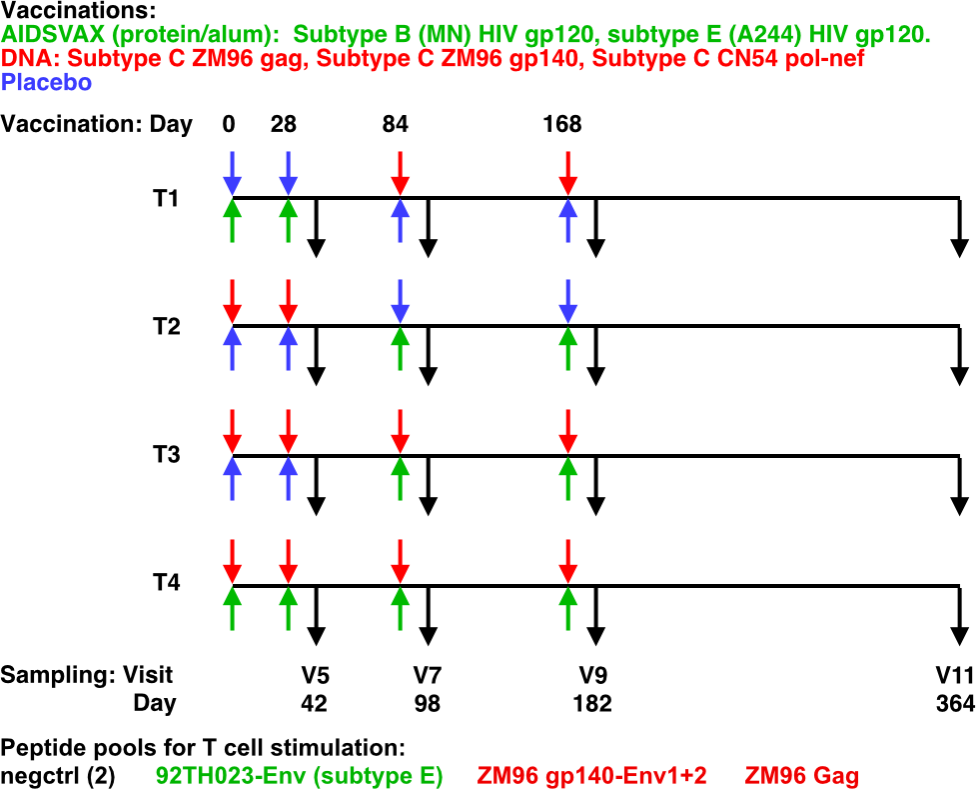
HVTN105 protocol. 26 participants per group were immunized with the indicated vaccines in the left (upper) and right (lower) deltoid muscles, at each of the time points indicated. Black arrows indicate PBMC sampling times.

The HVTN 105 trial administered intramuscular injections at 0, 1, 3, and 6 months (M). T1 received protein at M0 and M1 and DNA at M3 and M6; T2 received DNA at M0 and M1 and protein at M3 and M6; T3 received DNA at M0, M1, M3, and M6 with protein co-administered at M3 and M6; and T4 received protein and DNA co-administered at each vaccination visit (**Figure 1**).

The primary immunogenicity analysis was conducted 2 weeks following the final vaccination and evaluation of durability of the immune response was conducted at 6 months following the final vaccination. The previous analysis of humoral responses showed the groups receiving protein at M0 and M1, T1 and T4 had a >85% IgG response rate for ZM96.C and A244.AE after the second vaccination, however, the response rate for T1 was not sustained after subsequent vaccinations, likely a consequence of boosting with DNA only (2). After the final vaccination there was an 80% response rate for T2 and 100% for T3 and T4. Importantly, 2 weeks following the final vaccination, binding-IgG responses to the HIV V1V2 antigens that were identified as potential inverse correlates of risk (A244.AE V1V2 and 1086.C V1V2) from RV144 (3) were observed in 96% or more vaccinees in groups T2, T3, and T4. Over time geometric mean response magnitudes were similar across HIV antigens (vaccine-matched vs. consensus HIV envelopes, V1V2 antigens).

HIV-1–specific CD4+ and CD8+ T cell responses were examined by flow cytometry, using a validated 17-color intracellular cytokine staining (ICS) assay, two weeks after each boost as well as 12 months after enrollment. The peptide pools evaluated were vaccine matched (ZM96 gp140-Env1, ZM96 gp140-Env2, 92TH023-Env, and ZM96 Gag), covering Env and Gag. In the previous analysis, which was done with rigorous manual templated gating of the T cell populations, vaccine-induced CD4+ T cell responses were detected in all groups. There were minimal differences found across groups, although a trend of higher CD4+ responses in T3 was observed. However, this trend of higher CD4+ responses was significant when the polyfunctionality score was assessed (4). The two prominent polyfunctional CD4+ populations were the four-function IFN-γ^+^IL-2^+^TNF-α^+^CD40L^+^ and the three function IL-2^+^TNF-α^+^CD40L^+^.

The massive size and dimensionality of flow cytometry data is challenging for comprehensive manual analysis approaches, including its subjective and time-consuming nature, and the concern that novel and overlapping cell populations may be underappreciated with *a priori* gating strategies. Even at peak, there was an overall modest CD4+ T cell response rate to HIV Env (36%-60%) and low response to HIV Gag (0%-40%). We therefore re-analyzed the HVTN flow cytometry data, using the high-resolution SWIFT clustering algorithm (5, 6) that was originally developed to resolve rare cytokine-producing T cell subsets. We included batch registration (7) to reduce differences between batches that might obscure biological differences. Our goal was to increase the resolution of the heterogeneity of the T cell response, and to define responders more clearly.

It is anticipated that an effective HIV vaccine will require both optimal T cell and humoral immunity to confer protection. Given the inter-dependence of CD4+ T cell and antibody responses, we conducted correlative analysis of the clustered antigen-responsive T cell subsets with existing plasma antibody data sets to identify possible novel associations.

The re-analysis of flow data was consistent with the previous analysis, but yielded further discoveries of increased frequencies of participants with T cell responses; T cell sub-populations expressing IL-21; qualitatively different responses induced by DNA vs protein vaccines; and correlations between particular T cell subsets and subsequent antibody responses.

## Methods

### Data source

FCS files and de-identified metadata from intracellular cytokine staining (ICS) analysis of CD4+ and CD8+ T cell responses was provided from the HVTN from HVTN 105 a Phase 1 preventative vaccine trial (ClinicalTrials.gov NCT02207920). Primary ICS analysis was previously reported (2). Details regarding the study design, participants, sample and data acquisition are included in the primary study manuscript (2). Briefly, participants were randomly assigned to 1 of 4 groups with an allocation ratio of 1:1:1:1 (**Figure 1**). Participants received different combinations of AIDSVAX B/E, DNA-HIV-PT123, and placebo, administered intramuscularly. AIDSVAX B/E consisted of 300 μg of subtype B (MN) HIV gp120 glycoprotein and 300 μg of subtype A/E (A244) HIV gp120 glycoprotein adsorbed onto aluminum hydroxide gel adjuvant and administered into the right deltoid muscle. DNA-HIV-PT123 contained a mixture of 3 DNA plasmids: (a) clade C ZM96 gag, (b) clade C ZM96 gp140, and (c) clade C CN54 pol-nef, delivered at a total dose of 4 mg administered into the deltoid muscle via needle and syringe. Serum for humoral assays was obtained from serum-separating tubes (SSTs) and frozen at –80°C. Peripheral blood mononuclear cells (PBMCs) for cellular assays were isolated and cryopreserved from within 6 hours of venipuncture, as described previously (8). Flow cytometry was used to examine HIV-1–specific T cell responses using a validated intracellular cytokine staining (ICS) assay. The peptide pools evaluated were vaccine matched (ZM96 gp140-Env1, ZM96 gp140-Env2, 92TH023-Env, and ZM96 Gag), covering Env and Gag. Previously cryopreserved PBMCs were stimulated with the synthetic peptide pools. As a negative control, cells were not stimulated. Serum HIV-1–specific IgG, IgG3, IgG4, and IgA responses were measured with a custom HIV-1–binding antibody multiplex assay (BAMA) as previously described (9, 10) using gp120 proteins and V1V2 antigens detailed previously (11).

### Data transformation

The set of fluorescent dimensions ℱ in $z^{C}$ were transformed using the “log-like” inverse hyperbolic sine, sinh^-1^, in conjunction with a set of *F*-dimensional cofactors [*α*_1_, *α*_2_,…,*α_F_*] for each dimension j∈ℱ . Each vector $z_{j}^{C}$ was divided by its corresponding cofactor *α_j_* prior to transformation, which effectively removed the artifactual bimodality introduced by the raw sinh^-1^ transformation.

To determine a suitable set of cofactors, each vector $z_{j}^{C}$ was first transformed by sinh^-1^ (Equation 1) and its intensity histogram was examined.


(1) $$\sinh^{-1}z_{j}^{C}=\ln\left(z_{j}^{C}+\sqrt{1+z_{j}^{C}{\text{ 1}}^{2}}\right)$$

Each *α_j_* was defined as the hyperbolic sine, sinh, of half the magnitude of the distance between the positive *P* ^+^ and negative *P* ^-^ peaks (**Equation 2**) nearest zero in the intensity histogram of each $\sinh^{-1}z_{j}^{C}$ ,


(2) $$\begin{array}{l} \alpha_{j}^{T}=\frac{P^{+}-P^{-}}{2}+1\\ \alpha_{j}=\sinh\;\alpha_{j}^{T}=\frac{e^{\alpha_{j}^{T}}-e^{-\alpha_{j}^{T}}}{2} \end{array}$$

and because *P* ^+^ and *P* ^-^ were defined in the transformed space, sinh was required to convert values back to the raw data space. The cofactors were then applied as follows (Equation 3),


(3) $$z^{T}=\ln\left(\frac{z_{ij}^{C}}{\alpha_{j}}+\sqrt{1+{\left(\frac{z_{ij}^{C}}{\alpha_{j}}\right)}^{2}}\right)$$

Note that scatter dimensions are typically not sinh^-1^ transformed, but for convenience we refer to the full data (scatter included) after sinh^-1^ transformation simply as $z^{T}$ .

### Removal of saturated events

To identify saturated events, all raw data vectors ${\mathrm{Zj}}_{\;}$ were transformed by (**Equation 3**) with *α_j_* = 100 to yield $z_{j}^{T}$ . Then each $z_{\text{j}}^{T}$ was allocated to 1024 uniformly-spaced bins, denoted *s*_*bin*. Each minimum bin was defined by (Equation 4),


(4) $$s\_bin_{1j}=\sinh^{-1}\left(\frac{-2[\frac{\log_{2}R_{j}}{2}+2]}{100}\right)$$

and each maximum bin was defined by (Equation 5),


(5) $$s\_bin_{1024j}=\sinh^{-1}\left(\frac{R_{j}}{80}\right)$$

where *R_j_* was the channel-specific keyword-value range parameter ($PnR) from the TEXT section of the FCS file. To determine the saturated event threshold *h_j_*, we first examined a window *w_j_* of the top-most 61 bins (Equation 6),


(6) $$w_{j}=s\_bin_{\left[964,965,\ldots ,1024\right]j}$$

Then the median and robust standard deviation of the differences between consecutive bins were used to identify bins that contained extreme differences (Equation 7),


(7) $$\begin{array}{l} w_{j}^{D}=diff(w_{j})\\ w_{j}^{M}=median(w_{j}^{D})\\ w_{j}^{\sigma }=1.4826\times median(|w_{j}^{D}-w_{j}^{M}|)\\ w_{j}^{X}=w_{j}^{D}>(w_{j}^{M}+2w_{j}^{\sigma }) \end{array}$$

where *w^D^* was the difference between consecutive bins *w_b_* – *w_b_*_-1_ for b∈{2,3,…,61} , *w^M^* was the median difference, *w^σ^* was the robust standard deviation of differences, and *w^X^* was a vector of 1’s and 0’s that indicated the presence or absence (respectively) of extreme differences. If no extreme differences were found, the examination window was shifted by -1 bin, *w* = *s*_*bin*_[963,964,…,1023],_ and re-examined. This process was performed iteratively until at least 1 extreme difference was found. Then the lowest *s*_*bin_Xj_* that contained an extreme difference was identified by (Equation 8),


(8) $$X_{j}=min\left(argmax\left(w_{j}^{D}\circ w_{j}^{X}\right)\right)$$

and its corresponding histogram value $v_{j}^{T}$ was inverse-transformed back to a raw intensity by (Equation 9),


(9) $$\begin{array}{l} v_{j}^{T}=histogram\_value\left(s\_bin_{Xj}\right)\\ v_{j}=100\times \sinh\;v_{j}^{T} \end{array}$$

The saturated event threshold *h_j_* was set to the raw intensity *v_j_* (or 80% of the maximum data range, whichever was higher) as follows (Equation 10),


(10) $$h_{j}=max\left(v_{j},\;0.8\times R_{j}\right)$$

Finally, all events with raw intensities above the saturated event threshold were removed.

### Removal of time defects

To identify time defect events, corrected fluorescence data were sorted by time. Then each $z_{\text{j}}^{C}$ was allocated to *B n*on-uniformly-spaced bins, denoted *t*_*bin*, and each contained the same *bin*_*size* number of events as follows (Equation 11),


(11) $$\begin{array}{l} bin\_size=\{\begin{array}{c} 1000,\;N<100,000\\ 10,000,\;N>1,000,000\;\\ \frac{N}{100},\;otherwise \end{array}\\ B=\left[\frac{N}{bin\_size}\right] \end{array}$$

Then the median event value *m* was determined for each bin. The vector of bin median event values within each dimension *j* were Z-score standardized by (Equation 12),


(12) $$\begin{array}{c} {\tilde{m}}_{j}=\frac{1}{B}\sum_{b=1}^{B}m_{bj}\\ m_{j}^{\sigma }=\sqrt{\frac{1}{B-1}\sum_{b=1}^{B}|m_{bj}-{\tilde{m}}_{j}|}\\ m_{j}^{Z}=\frac{m_{j}-{\tilde{m}}_{j}}{m_{j}^{\sigma }} \end{array}$$

Then any bins containing time defects were defined by (Equation 13),


(13) $$D_{j}=\left|m_{j}^{Z}\right|>3$$

and all events within each $t\_bin_{Dj}$ were removed.

### Censored saturated events and time defects

The censoring process (described above) identified and removed:

1. raw fluorescent events that saturated above the limits of detection (saturated events).

2. corrected fluorescent events that contributed to inconsistent signals over time (time defects).

Following the removal of saturated events and time defects, new FCS files were generated from the remaining data. **Supplementary Figure 1** shows the number of cells per sample before and after censoring for all samples.

### New compensation matrices

The quality of compensation matrices was assessed in FlowJo, and any sub-optimal compensation values were manually corrected. The optimized compensation matrices were inserted into the FCS files.

### Modified channel names

All marker-fluor combinations were consistent across the entire dataset. However, some FCS files contained channel names that did not match other files. Any mismatched channel names were corrected, and new FCS files were generated.

### Batch registration

To remove variation due to experimental batches, while maintaining as much biological variation as possible, swiftReg (7) was used to register batches. This approach first registered each batch separately to the same reference batch, then applied the resulting batch-specific shifts to all individual samples in that batch.

To do this, first a SWIFT cluster template was produced from a concatenate of antigen-stimulated samples from a single reference batch, and similar concatenates from each of the other batches were registered by NDCR to the reference template. The resulting batch registration template contained batch-specific maps of cluster movement vectors that specify the value-adjustments necessary to bring that batch’s clusters into alignment with reference clusters. All individual samples in each batch were then registered using these batch-specific cluster movement vectors. This process generated new batch-registered FCS files.

### Debris removal

To enhance detection of rare, biologically-significant populations and reduce computational burden, all batch-registered samples were randomly sub-sampled and combined into a single concatenated FCS file that was then clustered by SWIFT. The resulting SWIFT cluster template was used to identify debris clusters in FSC-A and SSC-A, as well as non-CD4 T cells. New FCS files were generated from non-debris CD4+ events.

### Expanded select channel data

Detection of positive markers was selectively enhanced by smoothly increasing intensity values about a user-specified inflection point. The smooth increase was achieved by multiplying intensity values within a channel by a sigmoid function (**Supplementary Figure 2**) as follows (Equation 14),


(14) $$\begin{array}{l} r=\left[-3.00,-3.01,-3.02,\ldots ,3.00\right]\\ x=(r+1)\times L\\ y=normcdf\left(\frac{6}{P}r\right)\times (10^{w}-1)+1\\ s_{j}=interp1(x,\;y,\;z_{j}^{C},\;option)\\ z_{j}^{E}=s_{j}\circ z_{j}^{C} \end{array}$$

where *r* was a 1×601 vector of values between -3.00 and 3.00 with intervals of size 0.01, *P* was the degree of overlap in the expanded region (default *P* = 0.5), *L* was the user-specified inflection point where expansion occurred in that channel, *W* was the width of the expanded region in decades (default W=1 ), *s_j_* was a vector of scaling values that were multiplied with $z_{j}^{C}$ element-wise to produce expanded data $z_{j}^{E}$ , *normcdf* is a MATLAB function that returned a cumulative standard normal distribution, and *interp*1 is a MATLAB function that returned interpolated values of the function *y* = *f*(*x*) at specific query points $z_{j}^{C}$ by spline interpolation (*option* = 'spline').

### Aggregation of data

Because the Env-1-ZM96 and Env-2-ZM96 peptide pools constituted the non-overlapping peptides covering the Env-ZM96 Env sequence, the total Env-ZM96 response for each sample was calculated by combining Env-1-ZM96 + Env-2-ZM96 and subtracting negctrl1. Note that Negctrl1 was subtracted here once to account for the additional background contribution of combining raw Env-1-ZM96 + Env-2-ZM96 counts. The 92TH023-ENV samples were stimulated with peptides covering the whole 92TH023-ENV sequence (2).

The cell counts for AnyEnvNeg1 were then defined as the maximum of the cell counts for 92TH023-ENV or Env-ZM96 + Env-2-ZM96, minus the background from negctrl1. Because the 92TH023-ENV and ZM96 ENV sequences have some homology, it is very likely that some peptides, presented by the MHC alleles of some participants, will be cross-reactive between the two ENV peptide sets. However, the extent of cross-reaction cannot be estimated from this dataset, and so we used a conservative definition of the “Any-Env” response as the larger of the response against either ENV sequence. This uses the conservative assumption that all T cells cross-reacted, and therefore the total response is revealed by the higher of the two anti-Env responses.

The number of CD4+ T cells producing IL-2+ and/or IFNγ+ was expressed as a percentage of the total live cells in the corresponding sample. Percentages below 0.005 were thresholded to a minimum of 0.005.

### Identification of responders

To identify responders, the variance of cell counts was first stabilized across clusters. Cluster-specific scaling factors were defined as half the median of cell counts across all negctrl samples for each cluster, with low scaling factors thresholded to a minimum of 10. All counts were then transformed by inverse hyperbolic sine (asinh) after division by the cluster-specific scaling factor. Transformed stimulated counts (TSC) were obtained by subtracting each cluster’s transformed background count from its pairwise transformed stimulated count (for values above the threshold, this is analogous to a log ratio).

For each sample group defined by Treatment, Stimulation, Visit, and Cluster, the standardized pairwise background variances (SPBV) between Negctrl1 and Negctrl2 were defined as the square root of the sample-mean of the squares of their pairwise differences.

Then for each sample, the p-values (with a Null hypothesis of “no difference”) were determined by applying the normal distribution survival function (https://docs.scipy.org/doc/scipy/reference/generated/scipy.stats.norm.html) to the ratio of TSC over SPBV. This was performed separately for the stimulated sample with each background (Neg1 or Neg2). The final reported probability of a sample being a responder was then 1 minus the mean of its two p-values. All samples with a final probability of ≥98% were considered to be responders.

### Evaluation of antibody responses associated with T cell responses

Antibody levels measured by binding antibody multiplex assay (BAMA) for HVTN 105 were obtained from HVTN. To compare the CD4 responses to related antibody levels, the Spearman correlation coefficients were calculated for related antigens using log transformed antibody abundances. Specifically, CD4 responses upon ZM96 gp140-Env1 and ZM96 gp140-Env2 stimulations were compared with antibody responses to 96ZM651.D11gp120.avi, gp41, gp70–96ZM651.02 V1v2 antigens. CD4 responses to 92TH023-Env were correlated with antibody response to A244 gp120 gDneg/293F/mon, AE.A244 V1V2 Tags/293F and gp41 antigens. Finally, CD4 responses to ZM96 gag were compared with antibody responses to p24.

## Results

### Sample pre-processing

The HVTN 105 dataset comprised 3,200 .FCS files representing 24 batches, with accompanying compensation matrices for each batch. In general, the Visit 5 (V5), V7 and V9 PBMC samples for one participant were all analyzed in the same batch, whereas the V11 PBMC samples were analyzed in separate sets of batches. As described previously (2), if PBMC samples did not meet quality control criteria, those samples were re-analyzed in a subsequent batch, resulting in duplicate analyses. After curation according to these rules, the complete dataset potentially comprised four vaccine groups each containing 26 participants, eight *in vitro* antigen stimulations, and four time points, for a total of 3,328 flow cytometry samples. The study design did not include a placebo group receiving no HIV antigens, and the T cell data did not include a baseline sample, i.e. before vaccination. Therefore the important negative controls are the pairs of “negctrl” samples that did not receive *in vitro* stimulation with any antigens. Our re-analysis focused on six of the eight antigen stimulations: two negative control samples (negctrl2 and negctrl2); E92TH023_ENV; Env_1_ZM96; Env_2_ZM96; and Gag_ZM96. This resulted in a total of 2,496 potential samples. Due to some dropouts and missing negctrl replicates, the final number of flow cytometry samples in our re-analysis was 2,393. The samples, batches, repeated samples and final analyzed samples are shown in detail in **Supplementary Figure S3**.

A consensus .FCS file was produced by concatenating sub-samples of all HIV Ag-stimulated samples from all batches at the V9 time point. V9 was chosen because Visit 9 was the pre-determined immunogenicity time point for the HVTN105 trial, and for most groups and antigen stimulations *in vitro*, this was also the strongest response (see below). V9 samples were therefore enriched for the rare, activated T cells, facilitating capture of these cell populations in the cluster template. Because this concatenate included samples representing all HIV antigen stimulations, this is an objective way to include potential cell phenotypes induced by any of the HIV antigens in any treatment group.

**Figure 2:**
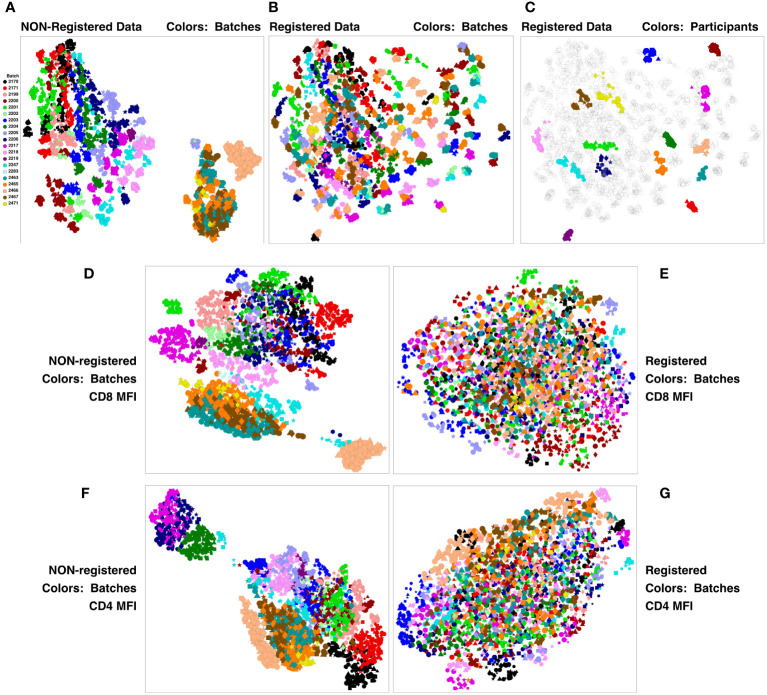
Registration minimizes batch variation and emphasizes individual stability. PBMC from the HVTN 105 vaccine trial from V5, V7, V9 and V11 (42, 98, 182 and 364 days) were analyzed by antigen stimulation, intracellular cytokine staining, and flow cytometry. A SWIFT cluster template was produced from a concatenate of HIV antigen-stimulated V9 (182 days) samples, then all individual samples were assigned to this template. All batches were then registered using swiftReg, and the registered samples were similarly analyzed by SWIFT clustering and individual sample assignment. For each of the original and registered datasets, all cluster information (sizes or MFI of individual parameters) was then condensed to two dimensions by UMAP. Each symbol represents one sample (one participant, one time point, one stimulation). Symbols: Circles, negctrl; triangles,E92TH023_ENV; stars, Env_1_ZM96; squares, Env_2_ZM96; and diamonds, Gag_ZM96. Symbol size, in increasing order, V5, V7, V9, V11. **(A-C)** UMAP plots represent all the numbers of cells/cluster information condensed down to two dimensions. **(A)** Unregistered, cluster sizes, batches colored. **(B)** Registered, cluster sizes, batches colored. **(C)** Registered, cluster sizes, 15 participants colored. **(D-G)** UMAP plots represent the UMAP condensation of the mean fluorescence intensities for each cluster of a specified marker. **(D, E)** CD4. **(F, G)** CD8. **(D, F)** Non-registered. **(E, G)**: Registered.

This concatenate was clustered using SWIFT to establish a high-resolution cluster template of all cell sub-populations. All samples were assigned to the resulting cluster template, establishing the number of cells in each cluster, in each sample. All cluster membership information was then condensed to two dimensions using UMAP (Uniform Manifold Approximation and Projection) (12). The results in **Figure 2A** show batches encoded by colors, stimulations by symbols, and visit number by symbol size. The strongest contribution to diversity was clearly the batch - most members of each batch are clustered together, and the batches are substantially resolved. This is particularly true for the V11 batches on the right, that were analyzed in a different set of batches from V5, V7 and V9. The presence of batch effects is not surprising in samples analyzed over a period of months - we have seen batch effects in all such datasets that we have examined. The HVTN105 batch effects were relatively minor, and so registration could be used to reduce batch effects and improve the comparison of the vaccine groups.

### Batch registration

We have previously developed swiftReg (7), an automated registration tool that builds on the SWIFT clustering algorithm to perform high-resolution alignment of samples at the single-cluster level. The HVTN 105 batches were registered by producing a SWIFT cluster template from Batch 2204, producing consensus samples from each batch, and then registering each batch consensus sample to the Batch 2204 consensus cluster template. This generated, for each batch, a map of registration shifts that were then applied to each individual sample in the respective batch. This procedure registers the overall batch trends, without altering the differences between individual samples within each batch that might carry biological information.

A new SWIFT cluster template was generated from a consensus of all registered V9 HIV antigen-stimulated samples. After assignment of all registered samples to the resulting cluster template, the cell numbers per cluster were reduced to two dimensions by UMAP, and **Figure 2B** shows that the registered batches were intermingled. ‘Micro-aggregates’ of samples from the same batch were still visible - focusing on just 15 participants for clarity, each micro-aggregate comprised samples from a single participant (including different stimulations and time points). These tended to group in close proximity on the UMAP projection (**Figure 2C**), even though the Visit 11 samples were analyzed in different batches from the Visit 5, 7, 9 samples. Overall, the samples included time points spanning 18 months. Thus, most individuals are sufficiently diverse for SWIFT analysis of flow cytometry data to identify a unique ‘fingerprint’ of cell populations in different participants. We have observed this pattern in other studies (unpublished). The proximity of the registered V11 data points to the V5, V7 and V9 points from the same participant reinforces the interpretation that the HVTN 105 batch effects have been substantially reduced by the registration process. Examination of individual parameters by the same approach identified parameters, e.g., CD4 and CD8, that contributed to these batch differences (**Figures 2D–G**). Interestingly, the groupings of similar batches were variable between different parameters (**Figure 2**; **Supplementary Figure 4**).

### Further pre-processing

We then used the model-based SWIFT multidimensional clustering algorithm (5, 6) to generate an unbiased cluster map from a sample constructed by concatenating a random subset of events from samples across all batches. The SWIFT algorithm is particularly useful for detecting rare populations (13), possibly because these were the type of samples used during SWIFT development (5, 6). Preliminary analysis of the cluster map indicated that the antigen-specific responses in many samples were small, consistent with the previous analysis (2). To maximize the sensitivity of detecting all cytokine producing sub-populations, we produced a new SWIFT cluster template from a concatenate of random subset of events from all antigen-stimulated samples, using only the scatter, live/dead, and CD4 parameters. All samples were assigned to the resulting template. As described previously (2), the non-replicating vaccines induced almost no CD8 T cell responses, and so further analysis focused on CD4 T cells. Clusters containing CD4 T cells were selected, and all the events in this set of clusters were saved, for each flow cytometry sample, as reduced-size .FCS files for further analysis. This “cluster gating” (6) allowed subsequent analysis to focus more clearly on the cells of interest, because clustering could then be performed on a full concatenate of the entire dataset. The resulting .FCS files are more amenable to analysis by SWIFT, other automated algorithms, and manual analysis.

### High-resolution clustering

A large concatenate was then produced from all cluster-gated events in all samples stimulated with HIV antigens (Env, Gag), from all groups at Visit 9, which was the time point that showed the highest responses overall. A second concatenate was produced from the corresponding negative control samples. SWIFT cluster templates were created from each of these two large concatenates, using all parameters for high-resolution clustering. The two resulting cluster templates were combined, and all individual samples from all groups, all visits, all stimulations were assigned to the resulting combined template (total clusters 2,246). This cluster competition approach (7) sharpens the differences between the two groups represented by the two templates, in this case stimulated and unstimulated cell populations. Note that each concatenate included samples from all vaccine groups, so the competition process should not affect the resolution or statistical analysis of any study group differences.

**Figure 3:**
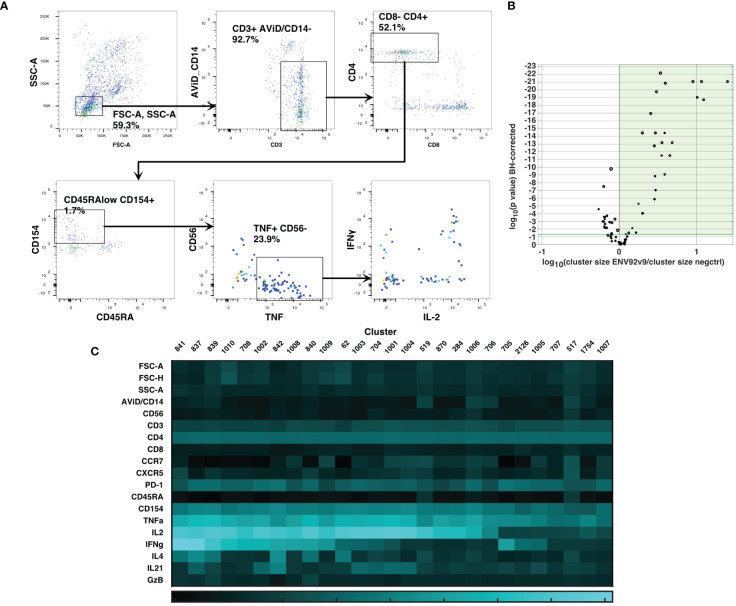
Cluster gating of cytokine-producing antigen-specific T cells. SWIFT cluster templates were produced from concatenates of antigen-stimulated samples, and control samples, and the two templates combined for competitive cluster assignment. All individual samples were assigned to the combined template. **(A)** All cells were plotted at their cluster medians in each parameter for cluster gating on bivariate plots, to identify activated CD4 T cells expressing CD154 and TNF. **(B)** For each cluster, the number of cells in a concatenate of ENV92-stimulated visit 9 samples was compared by Wilcoxon to the matched negative control sample. Each symbol indicates one cluster, and the size of the symbol is proportional to the mean number of cells per cluster. P values were adjusted according to the Benjamini-Hochberg method for multiple measures. The green shaded area indicates the clusters that were significantly increased in size by antigen stimulation. **(C)** The heatmap shows the median fluorescence intensity in each parameter (Z-scores) of the 27 significantly induced clusters from B (shaded area).

Cluster gating (6) was then used to narrow down the cell populations of interest. During cluster gating, all cells are assigned their cluster medians in all dimensions, so that the two-dimensional gating shown in **Figure 3A** takes advantage of all the information in all dimensions. Activated CD4 T cell clusters were identified as live, singlet, CD3+ CD4+ CD154+ TNF+ clusters (**Figure 3A**). Additional marker intensities for all parameters are shown in **Supplementary Figure S5**. These activated CD4 T cell clusters were then examined by testing the significance of differences between antigen-stimulated and negative control clusters in all participants at visit 9. A Wilcoxon test was followed by the Benjamini-Hochberg correction for multiple measurements, because of the number of clusters examined. **Figure 3B** shows the ratios and magnitudes of differences between antigen-stimulated and negative control cultures in a volcano plot. All clusters that were significantly increased in the antigen-stimulated samples (green shaded area) were chosen for further analysis. To facilitate comparisons with previous analysis (2), the SWIFT clusters were aggregated into four groups: IL-2+IFN-γ+, +/-, -/+ and -/- cells (15, 5, 3 and 4 clusters, respectively). The heatmaps (**Figure 3C**) show the marker characteristics of each cluster.

### Identification of vaccine responders

**Figure 4:**
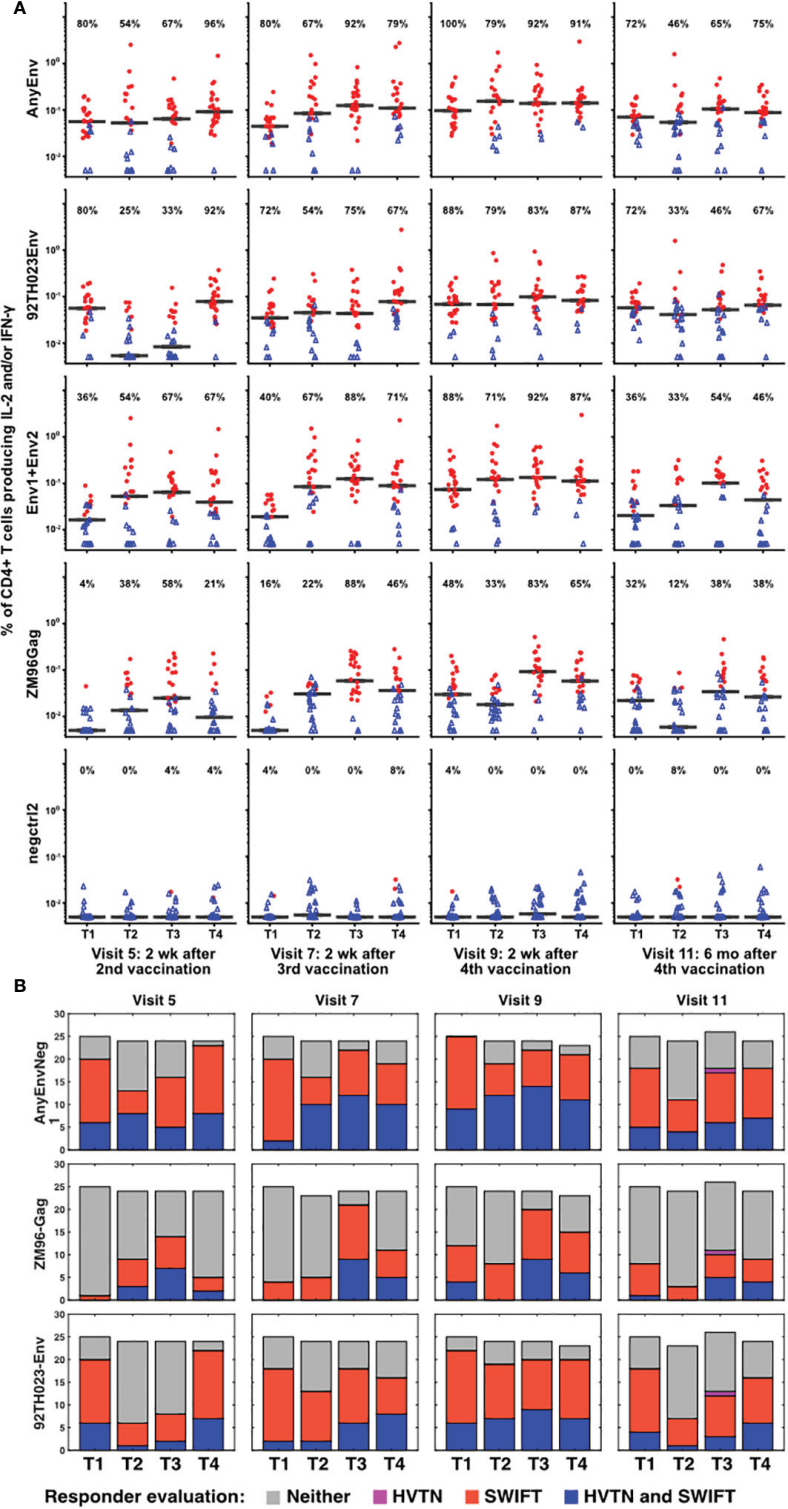
Increased numbers of vaccine responders identified by detailed analysis pipeline. **(A)** For all V5, V7, V9 and V11 samples, responders were identified as described in Methods, calculating the responses separately for 92TH023 Env; ZM96 pool 1 + pool 2 Env; ZM96 Gag; the negative control negctrl2; and Any Env (the larger of the responses to either 92TH023 or ZM96 Env1 + Env2). The values from negctrl1 were subtracted from each of these values. Red circles and blue triangles indicate responders and non-responders, respectively, and horizontal black bars indicate medians of all samples in each treatment group. The percentage of positive responses is shown above each graph. Values less than 0.005% were plotted at 0.005%. **(B)** Responder rates from the present study compared to the equivalent responder rates from the original analysis (2).

The samples showing significant responses to each antigen, at each time point, were then evaluated as described in Methods, using the aggregated cluster data for all clusters producing IL-2 and/or IFN-γ. **Figure 4** shows the results for each time point, each vaccine treatment, and five antigen stimulations, or combinations of stimulations: AnyEnv (Env92 or Env1/2), Env92 (Env92TH023 only), Env1/2 (Env1 plus Env2), GAG-ZM96, and the negctrl2. Background values (negctrl1) were subtracted from all antigen-stimulated values (similar conclusions were obtained if the negative controls were reversed). All samples with >98% probability of being genuine responders are shown in red. As expected, very few negctrl samples were evaluated as responders (at a confidence level of 98%, a small number of false positives are expected). As Gag antigen was only included in the DNA vaccine, Treatment 1 uniquely lacks immunization with Gag for the first blood sample evaluated, at Visit 5. Consistent with this, only Treatment group 1 lacks a response to Gag at Visit 5. At a very high confidence level of 99.9%, there were still high rates of responders (up to 88%) but no responders in any negative controls (**Supplementary Figure S6**). **Supplementary Figure S7** shows an alternative layout of the responder data to emphasize the time course within each group.

Several combinations of vaccine treatments and times induced responses in the great majority of participants, particularly in Treatment group 3 at Visits 7 and 9. The numbers of responders were generally higher than evaluated previously (2), possibly because the extensive pre-processing and the competitive cluster templates used in our analysis provided sharper distinction between antigen-stimulated versus background cells producing cytokines. The magnitude of the net anti-HIV T cell responses was well-correlated between the original analysis and the re-analysis (**Supplementary Figure S8**). There is a general trend towards higher magnitudes detected by SWIFT (compared to the 1:1 reference line), possibly due to the effectiveness of high-dimensional definition of populations, as well as the sharper signal:noise discrimination by focusing on the clusters that were significantly increased by antigen stimulation.

### Qualitatively different responses are associated with different vaccine modalities

**Figure 5:**
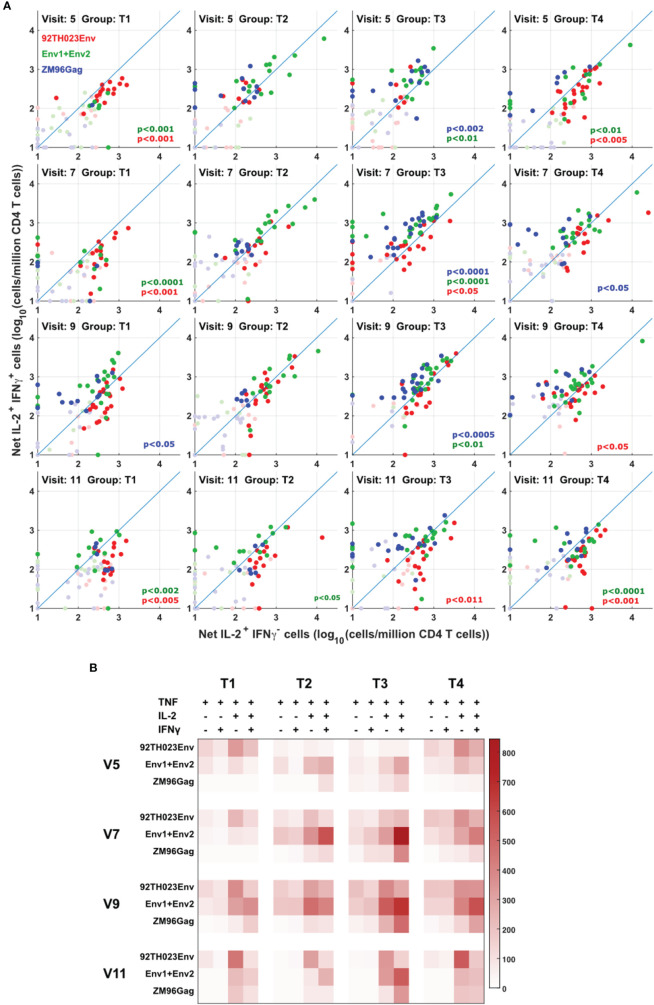
Different cytokine response patterns associated with DNA or protein vaccination. **(A)** IL-2+IFNg+ responses were compared with IL-2+IFNg- responses in all participants, all visits and for ZM-96-Gag, ZM96-Env and 92TH023-Env. Dark symbols indicate samples with positive responses (using the values for IL-2 and/or IFNγ from **Figure 4**) and pale symbols indicate non-responders. P values indicate the significance of the deviation from the 1:1 correlation line, with colors matching the data points. **(B)** The heatmap indicates the average number of antigen-responsive CD4 T cells per million total live CD4 T cells, for each vaccination group. Each response is divided into all combinations of IL-2 and IFNγ expression.

The quality of the cytokine response to protein or DNA-derived immunogens was assessed between the different vaccine treatments by comparing the ratio of T cells producing IFN-γ vs. T cells producing IL-2 but not IFN-γ. The anti-Gag response is easiest to interpret, as this is induced only by the DNA vaccine. **Figure 5A** shows that this response is biased towards IFN-γ production, consistent with a previous report (14). The response to the ZM96 clade C peptides, primed by DNA, also showed a tendency towards an IFN-γ-biased response. In contrast, the response to clade E 92TH023 protein immunization was biased more towards IL-2-only responses, consistent with our previous demonstration (15) that viral infections tend to induce more Th1/IFNγ responses, whereas protein vaccines tend to produce responses biased towards IL-2-producing central memory (16) cells. **Figure 5B** summarizes these results, including the results for the minority IL-2- IFN-γ- and IL-2- IFN-γ+ responses.

### Minority cytokine responses

**Figure 6:**
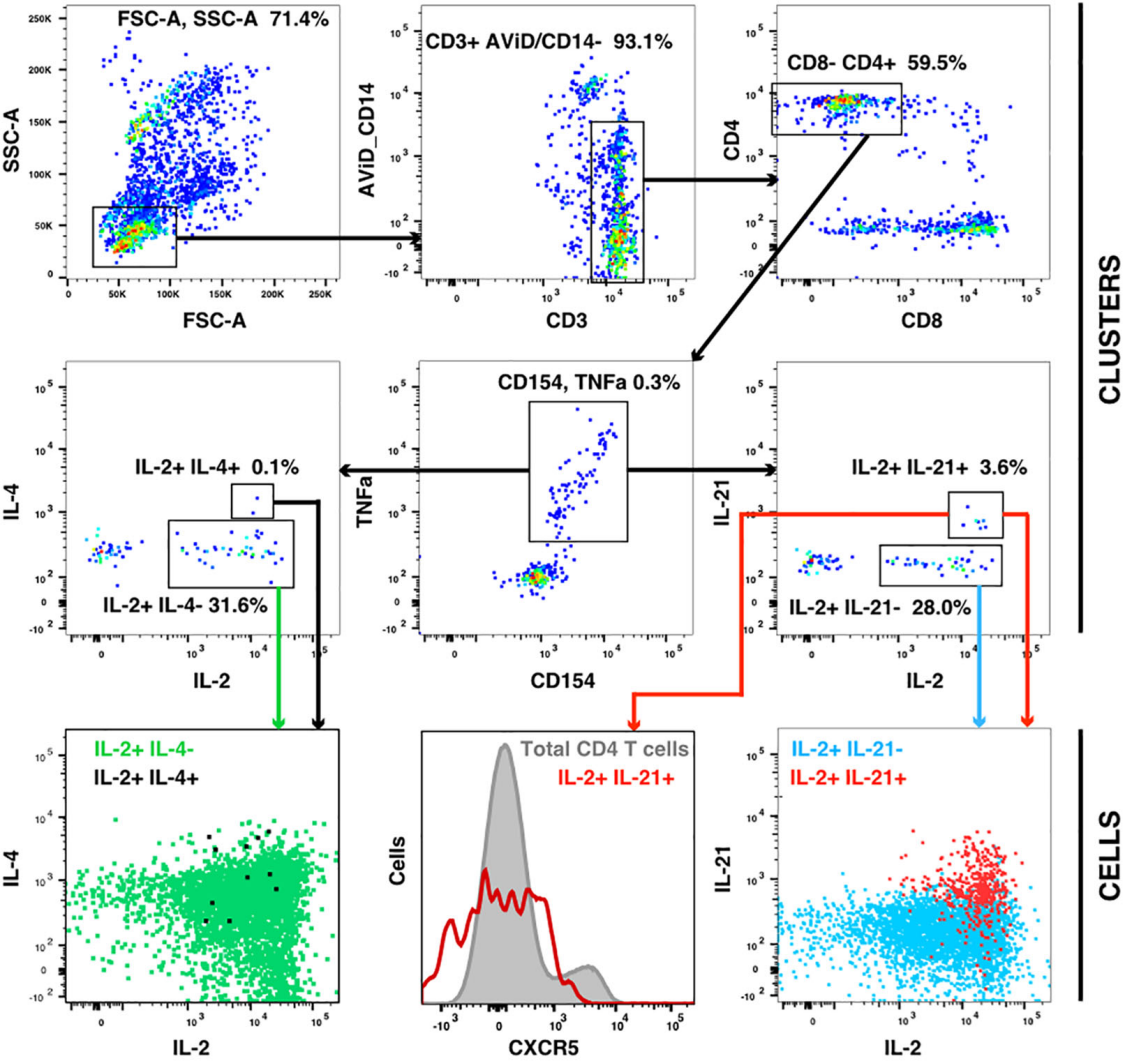
IL-21 responses after vaccination. A concatenate (10 million cells) of random samples of all HIV antigen stimulated samples at V9 was assigned to the cluster template used in **Figure 3**, and cluster gating was used to identify all CD4+ CD154+ TNF+ T cells (center panel). Cluster gating was used to further identify IL-2+ IL-4+ and IL-2+ IL-4- clusters (second row, left) and IL-2+ IL-21+ and IL-2+ IL-21- clusters (second row, right). In the top and middle panels, each dot represents one cluster. The bottom row shows plots of individual cells in the four sets of clusters.

**Figure 7:**
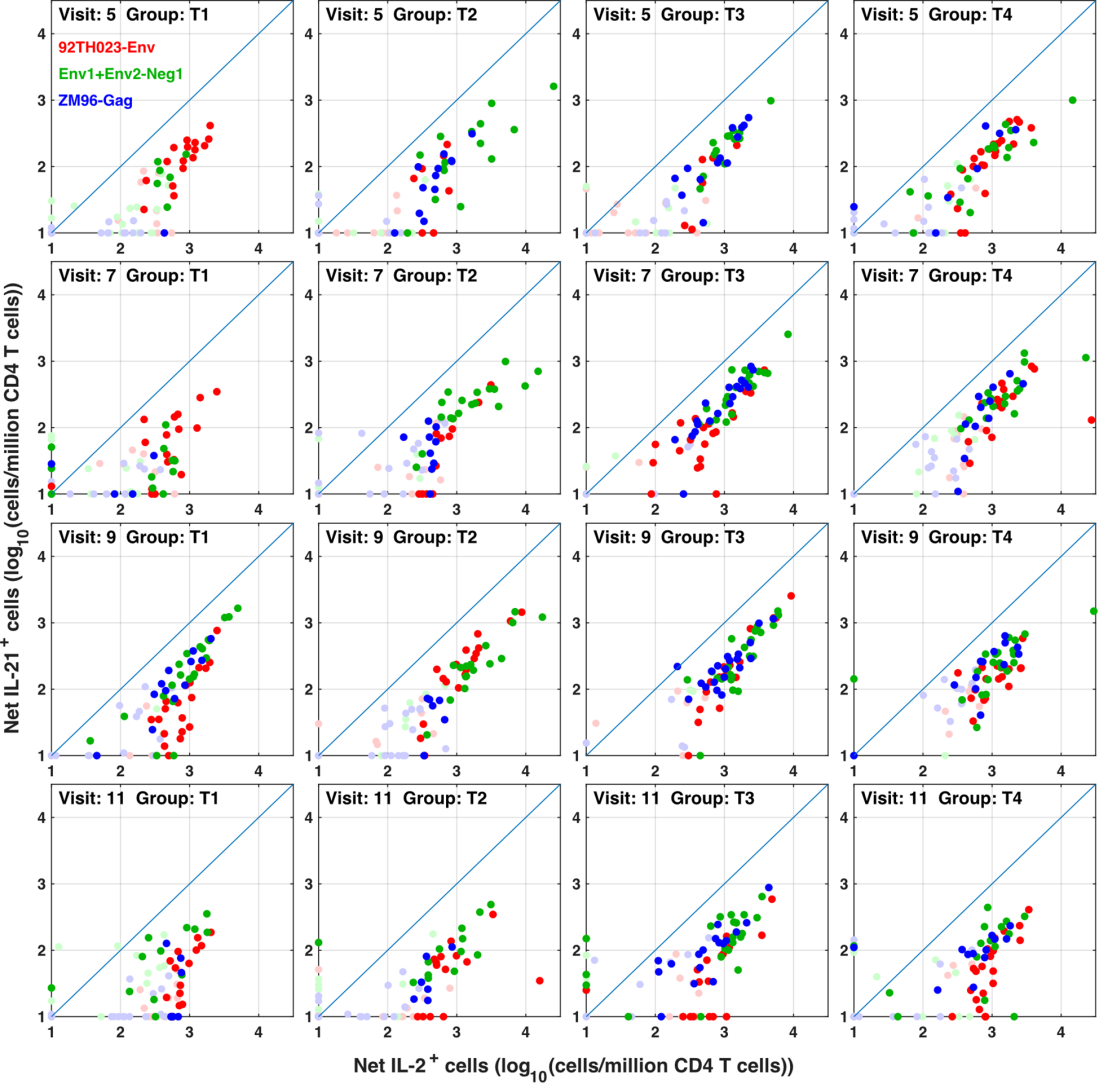
IL-21 responses to different immunogens. CD4 T cells producing IL-2 (with or without IFN γ) were compared with IL-21+ responses in all participants, all visits and for ZM-96-Gag, ZM96-Env and 92TH023-Env antigen stimulations. Dark symbols indicate samples with positive responses (using the values for IL-2 and/or IFNγ from **Figure 4**) and pale symbols indicate non-responders.

The flow cytometry panel included several cytokines, including IL-21 (produced by Tfh and some other cells) and IL-4 (produced by Th2 cells). Manual examination of the concatenated results suggested that antigen stimulation appeared to induce a small IL-21 response in a relatively low number of TNFa+ IL-2+ CD4 T cells. However, the IL-21 staining was weak, and did not result in a clearly separated sub-population of positive cells. As the SWIFT clustering algorithm uses a criterion of multidimensional unimodality to define individual sub-populations (6), the putative IL-21+ cells were initially difficult to identify by clustering. We therefore used a ‘stretching’ modification that slightly broadened the cell distribution across the expected junction between IL-21- and IL-21+ cells. Clustering the resulting data in SWIFT allowed the reproducible detection of IL-21+ clusters (**Figure 6**). In contrast, applying the same stretching modification to the IL-4 channel did not result in the detection of IL-4+ clusters, consistent with the manual examination of the IL-4 data (**Figure 6**). The IL-21+ clusters were activated memory CD4 T cells (CD154+ CD45RAlo CD4+), but interestingly, did not express the CXCR5 chemokine receptor that is characteristic of circulating T follicular helper (Tfh) cells (**Figure 6**), perhaps due to down-regulation of CXCR5 on the *in vitro* activated cells as we have observed previously (S. De Rosa, unpublished). In contrast to the IL-2+ IFN-γ+ versus IL-2+ IFN-γ- skewing described above, the IL-21+ cells were observed in all treatment groups, and did not show obvious biases towards particular antigens or immunization strategies (**Figure 7**).

### Correlations of SWIFT CD4+ T cell clusters with HIV-specific plasma antibody responses

**Figure 8:**
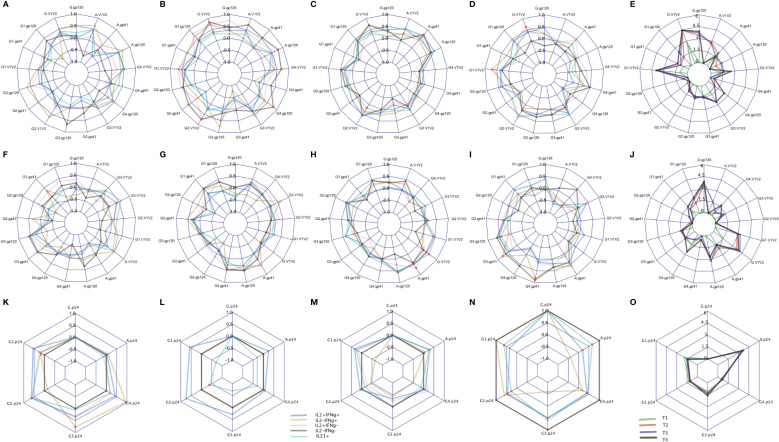
CD4 responses associated with antibody levels measured by binding antibody (BAMA) assay. The spider plots **(A–D, F–I, K–N)** show correlation coefficients between CD4 clusters (blue: IL2+IFNℽ+, yellow: IL2-IFNℽ+, pink: IL2+IFNℽ-, grey: IL2-IFNℽ- and aquamarine: IL21+) and antibody levels across four treatment groups. The spider plots **(E, J, O)** show log-transformed magnitudes of antibody levels (T1: Green, T2: red, T3: blue, T4: Black). The top, middle and bottom plots show BAMA correlations to CD4 responses to Env92 (gp120-A244 gp120 gDned/293F/mon, V1V2- A244 V1V2 Tags/293F; A,B, C,D), Env1 + 2 (gp12–96ZM 651.D11gp120.avi, V1V2–96ZM651.02 V1V2; F,G,H,I) and Gag (K,L,M,N). Red dots indicate significant correlations with adjusted p-value<0.05.

Binding antibody (Ab) responses were the major correlates of risk (CoR) identified in the RV144 Trial (3). Subsequently, at V9, 2 weeks after the final immunization we assessed the relationship of IL-2/IFN-γ and IL-21 defined clusters with the contemporary HIV-specific plasma Abs (**Figure 8**). Overall T1 (**Figures 8E, J**) had low antibody responses at this timepoint compared to the other groups as expected due to the boosting immunizations with DNA alone. T2 overall exhibited the greatest number of significant correlations with the IgG response, primarily associated with responses to AE.A244 (**Figure 8B**) which most closely matches the protein component of the vaccine regimen. T3 and T4 overall had significant associations relatively balanced between AE.A244 (**Figures 8C, D**) and 96ZM651 (**Figure 8H**) Ab responses which most closely matches the DNA component of the vaccine regimen, and is consistent with T3 and T4 receiving 4 doses of DNA. T3 had the greatest number of significant correlations between IL-21+ and Ab responses (**Figures 8C, H**), consistent with T3 having the overall greatest IL-21+ response. **Supplementary Figure S9** shows the magnitude of IgG responses for the T cell responders identified by the original analysis or the new SWIFT analysis.

Both total IgG specific for the V1V2 region of gp120 and IgG3 specific for V1V2 were inverse CoR in RV144 (3). IL-2-IFN-γ+ cells were significantly correlated with IgG AE.A244 V1V2 in T4 (**Figure 8D**), with IL-2+IFN-γ+ and IL-2-IFN-γ also significantly correlating with IgG AE.A244 V1V2 in T2 (**Figure 8B**). IL-2+IFN-γ+ also, and IL-21+ also significantly correlated with IgG gp70–96ZM51 V1V2 in T3 (**Figure 8H**). V1V2 responses of the specific IgG subclass, IgG3 which is known to be a potent mediator of Fc-effector functions such as antibody dependent cellular cytotoxicity were inverse CoR in RV144, and only IL-2+IFN-γ+ in T1 was significantly correlated with IgG3 gp70–96ZM51 V1V2 (**Figure 8F**). IgA specific for gp120 overall as well as the V1V2 region was a CoR in RV144, suggested to compete with the binding of protective IgG3 (3, 17). Only T2 and T3 had measurable IgA responses to AE.A244 gp120 or AE.A244 V1V2 (**Figure 8E**), with IL-2-IFN-γ- in T2 significantly correlating with IgA AE.A244 gp120 and IgA AE.A244V1V2 (**Figure 8B**). IL-21+ in T3 was significantly correlated with both IgA AE.A244 gp120 and IgA AE.A244V1V2 (**Figure 8C**). Overall these results indicate subtleties in the association of CD4+ T cell responses and plasma Ab responses, that are impacted by vaccine regimen and may provide insight into efficacy outcomes.

## Discussion

A substantial preventative vaccine trial such as HVTN 105 generates a large dataset of immunological results, which provides a valuable resource for continued analysis using different approaches. This trial was chosen for analysis, although a phase I trial, because it reiterated the general prime-boost approach of the only preventive vaccine trial to show any degree of efficacy, RV144 (“The Thai Trial”), but with priming by a more flexible DNA vaccine platform. We have re-analyzed the flow cytometry T cell response data using a detailed clustering approach, and also evaluated the correlations between different T cell responses and the levels of different isotypes and specificities of antibodies. This resulted in the detection of higher numbers of responders; revealed preferential induction of central versus effector T cell responses by different immunogens; and showed that the best correlations between T cell and antibody responses did not necessarily match the strongest responses.

The SWIFT clustering algorithm is highly effective for detecting small cell sub-populations in flow cytometry data (6, 18). This sensitivity may be related to the extensive use of antigen-stimulated PBMC datasets during SWIFT development, resulting in an algorithm that is well-suited to the detection of small cytokine-producing T cell responses of human PBMC, e.g., in the HVTN 105 dataset.

An additional advantage of the SWIFT analysis pipeline is the registration tool swiftReg (7), which can register batches of data to minimize batch effects while preserving biological variation and group differences. The HVTN 105 trial was large, and the flow cytometry data analysis was performed in many batches. Although stringent protocols ensured that the batch variation was smaller than in many other studies, it is almost impossible to completely prevent batch effects in experiments conducted over several months, and so the swiftReg tool was helpful in minimizing batch variation to allow the analysis to focus more sharply on the vaccine group differences. As swiftReg produces new .FCS files containing registered data, registration can also be a useful step in data processing pipelines using alternate clustering approaches.

Compared to the initial analysis (2) the high-resolution SWIFT analysis detected substantially higher numbers of responding participants for all antigens. A major contribution to this increase may have been due to our sharpened discrimination of responders from non-responders using competitive template assignment (19). In this approach, SWIFT cluster templates were produced from two concatenates, of antigen-stimulated and negative control samples. These two templates were then combined and all samples assigned to the joint template. Some cytokine-secreting clusters preferentially captured background responses, so by focusing only on clusters that were significantly higher in antigen-stimulated samples, we were able to sharpen the identification of antigen-responding cells and improve signal:noise ratios. This probably contributed to the higher number of responders detected, while the overall pattern of the response was similar, e.g., group T3 had higher responder frequencies in both analyses.

Several issues have to be considered for the potential T cell cross-reactions between different antigens used in the HVTN105 study. The predictions for anti-Gag responses are relatively straightforward, because Gag antigens were encoded by the DNA vaccine, but not included in the protein vaccine. Thus Gag responses should be attributable only to Gag-ZM96 priming and boosting. Consistent with this prediction, significant numbers of Gag responders were only observed in groups that had received the DNA vaccine prior to the sample draw. In addition, Gag responses are simpler to interpret because the immunogen and the *in vitro* challenge peptides were fully matched.

In contrast, three different Env sequences were included in the vaccines. The DNA vaccine expressed the clade C ZM96 gp140 protein, whereas the protein AIDSVAX vaccine contained both the clade B gp120 MN and clade E gp120 A244 proteins. Thus, the DNA and protein vaccines should stimulate partially overlapping T cell repertoires specific for Env, and a second immunization with the other vaccine type (protein to DNA, or DNA to protein) should induce a mixture of memory responses to cross-reactive epitopes, and naïve responses to non-cross-reactive epitopes.

*In vitro* testing of T cell anti-Env responses was performed with three peptide pools: Two vaccine-matched peptide pools covered the N-terminal and C-terminal regions of the ZM96 clade C gp140 protein, and a third pool contained peptides of the clade E 92TH023 protein, i.e. the same clade as the AIDSVAX clade E Env A244 protein, but with only about 90% homology between the protein sequences. However, the two proteins contain long stretches of completely homologous sequences, so there should be substantial but not complete cross-reaction between the immunizing and testing clade E Env epitopes. Responses to the immunizing clade B MN env protein would be expected to have lower cross-reactivity to either the clade C or Clade E test antigens, and so may not have contributed significantly to the overall *in vitro* T cell results. Because the extent of cross-reaction between the clade C- and clade E-specific T cells in this study was unknown, we made the conservative assumption that the “any env” response was taken as the maximum of the ZM96 and 92TH023 responses, i.e., assuming complete cross-reaction, as in the previous analysis (2).

The quality of the T cell response, i.e. the cytokine patterns produced by antigen-specific T cells, was influenced by the type of vaccine. In line with previous studies (14, 15) the AIDSVAX protein vaccine preferentially induced CD4 T cells producing IL-2 but not IFNγ, whereas the DNA vaccine induced more IL-2+ IFNγ+ T cells. The IL-2+ cells may be central memory T cells (Tcm) (16) that have high proliferative potential and can differentiate into effector cells (16, 20), whereas the IL-2+ IFN-γ+ T cells are effector memory cells. While both T cell populations are potentially valuable for future protection, the Tcm may have higher potential over longer times (21).

In addition to the evaluation of the major cytokines TNF, IFN-γ and IL-2, the flow cytometry analysis also measured IL-21-producing cells. Although the staining for IL-21 was not strong, there appeared to be an IL-21+ population that expressed high levels of TNF and IL-2, and variable amounts of IFN-γ. IL-21 is produced commonly, although not exclusively, by CXCR5+ Tfh cells in lymph nodes (22, 23). However, the IL-21+ cells in the HVTN105 study were generally CXCR5-. Although this might suggest that these were not circulating Tfh-like cells (24, 25) it is also possible that CXCR5 expression was lost during *in vitro* stimulation.

Assessment of the SWIFT-defined CD4+ T cell clusters’ association with the plasma Ab response to HVTN 105, revealed that although polyfunctional TNF-alpha+ IL-2+ IFN-γ+ effector memory cells dominated the CD4+ T cell response in T3 and T4, a subdominant IFN-γ producing population, IFN-γ+IL-2- cells in T3 and T4 correlated with IgG AE.A244 V1V2 (an inverse CoR in RV144), suggesting that the magnitude of a specific CD4+ T cell cluster is not the sole determinant of correlation with the Env-specific Ab response. The consequences of associations between CD4+ T cell cytokine producing subsets and protective antibody responses to HIV remain uncertain, however intriguing findings regarding this relationship continue to emerge.

A limitation of this study was that although HVTN 105 used the same protein immunogen as RV144, AIDSVAX B/E, unlike RV144, HVTN 105 was not an efficacy trial. Subsequently, the differences observed in response rates or phenotypes of CD4+ T cells observed between groups in HVTN 105 either in this re-analysis or the primary analysis (2) cannot infer association with vaccine efficacy.

The recent HVTN 702 efficacy trial conducted in South Africa, which was an iteration of RV144 with Clade C immunogens consisting of priming with a canarypox-based env/gag/pro immunogen and boosting with the addition of a Env protein immunogen, unfortunately resulted in similar infection rates in placebo and vaccine recipients (26). *Post-hoc* analysis revealed that among individuals that had high IgG AE.A244V1V2 responses, CD4+ T cell polyfunctional score was associated with lower risk of HIV acquisition. And conversely, among individuals that had low IgG AE.A244V1V2 responses, the CD4+ T cell polyfunctional score was associated with a higher risk of HIV acquisition. These findings highlight the increasing need to better define and monitor the nuanced relationship between the CD4+ T cell response to HIV vaccines and the protection that may be conferred by antibody responses, and we suggest that advanced flow cytometry analysis approaches, such as SWIFT, can enhance resolution of the HIV-specific T cell response.

## Software availability

The SWIFT and swiftReg software packages are freely available for download at: http://www.ece.rochester.edu/projects/siplab/Software/SWIFT.html.

## Data availability statement

The original contributions presented in the study are included in the article/**Supplementary Materials**, further inquiries can be directed to the corresponding author/s.

## Ethics statement

Ethical approval was not required for the studies involving humans because the samples came from a previously published clinical trial (ClinicalTrials.gov NCT02207920). The studies were conducted in accordance with the local legislation and institutional requirements. Written informed consent for participation was not required from the participants or the participants’ legal guardians/next of kin in accordance with the national legislation and institutional requirements because samples were de-identified prior to the analyses.

## Author contributions

TM: Writing – review & editing, Writing – original draft, Visualization, Validation, Supervision, Software, Project administration, Methodology, Investigation, Funding acquisition, Formal analysis, Data curation, Conceptualization. JR: Writing – original draft, Software, Methodology, Investigation, Formal analysis, Conceptualization. SR: Writing – review & editing, Resources, Investigation. MK: Writing – review & editing, Project administration, Investigation, Funding acquisition. MM: Writing – review & editing, Resources, Investigation, Funding acquisition. NR: Writing – review & editing, Resources, Investigation, Funding acquisition. GP: Resources, Investigation, Writing – review & editing. PG: Writing – review & editing, Resources, Investigation. LC: Writing – review & editing, Resources, Investigation. JK: Writing – review & editing, Writing – original draft, Supervision, Project administration, Funding acquisition, Conceptualization. JT: Writing – review & editing, Writing – original draft, Supervision, Software, Resources, Methodology, Investigation, Formal analysis, Data curation, Conceptualization.
